# A promising new wavelength region for three-photon fluorescence microscopy of live cells

**DOI:** 10.1111/j.1365-2818.2012.03610.x

**Published:** 2012-06

**Authors:** Greg Norris, Rumelo Amor, John Dempster, William B Amos, Gail McConnell

**Affiliations:** * Centre for Biophotonics, SIPBS, University of Strathclyde161 Cathedral Street, Glasgow G4 0RE, United Kingdom; † MRC Laboratory of Molecular BiologyHills Road, Cambridge CB2 0QH, United Kingdom

**Keywords:** Multi-photon, live cell, cytoplasmic streaming

## Abstract

We report three-photon laser scanning microscopy (3PLSM) using a bi-directional pumped optical parametric oscillator (OPO) with signal wavelength output at λ= 1500 nm. This novel laser was used to overcome the high optical loss in the infrared spectral region observed in laser scanning microscopes and objective lenses that renders them otherwise difficult to use for imaging. To test our system, we performed 3PLSM auto-fluorescence imaging of live plant cells at λ= 1500 nm, specifically *Spirogyra*, and compared performance with two-photon excitation (2PLSM) imaging using a femtosecond pulsed Ti:Sapphire laser at λ= 780 nm. Analysis of cell viability based on cytoplasmic organelle streaming and structural changes of cells revealed that at similar peak powers, 2PLSM caused gross cell damage after 5 min but 3PLSM showed little or no interference with cell function after 15 min. The λ= 1500 nm OPO is thus shown to be a practical laser source for live cell imaging.

## Introduction

Scanning microscopy using two-photon excitation of fluorescence is well established. However, it is desirable to explore the potential of three-photon processes, which are relatively little studied. It may be that researchers have been deterred by the requirement for higher peak intensities predicted from theory for three-photon excitation (Xu *et al.,* 1996), and fear of heating of the specimen because of higher absorption in aqueous media (Träutlein *et al.,* 2008). However, a possible advantage of using three photons rather than two is that longer wavelengths can be used, possibly with less cytotoxic effect, and the resolution loss that results from the longer wavelength can be to some extent reversed by the intensity-cubed dependence of the three-photon excitation process (Gu, 1996).

Some promising results have already been obtained for three-photon laser scanning microscopy (3PLSM) using third harmonic generation (Barad *et al.,* 1997; Mueller *et al.,* 1998; Squier *et al.,* 1998; Millard *et al.,* 1999; Canioni *et al.,* 2001) For example, by increasing the wavelength to λ= 1260 nm with a femtosecond-pulsed Cr:Forsterite laser, an improved depth of tissue imaging by third harmonic generation has been achieved (Debarre *et al.,* 2006; Sun *et al.,* 2004) and three-photon excitation laser scanning fluorescence has been demonstrated (Matsuda *et al.,* 2006) for a range of samples, albeit at shorter (λ < 1100 nm) wavelengths. It is significant that the peak intensities used for 3PLSM in these reports were similar to those routinely used in two-photon laser scanning microscopy (2PLSM), and excessive damage to the specimen was not seen.

For three-photon excitation of fluorophores with single-photon absorption in the visible region of the spectrum, wavelengths are needed which are above the range of the ubiquitous Ti:Sapphire laser (Wokosin *et al.,* 1996) and the Cr:Forsterite laser, so, in this work, we have examined excitation at λ= 1500 nm. Unfortunately, few suitable lasers exist with such long-wavelength emissions. A fibre laser has been used (Millard *et al.,* 1999) to record third-harmonic images from plant leaves, but fluorescence emission was not studied. The fibre laser is also fixed wavelength, which is a drawback for fluorescence excitation. Wavelength tuning in the required spectral region is most frequently achieved using expensive commercial optical parametric oscillators (OPOs) synchronously pumped by a similarly high-cost femtosecond-pulsed Ti:Sapphire laser. However, the frequency conversion process is often inefficient and the average power from these systems at λ= 1500 nm is often too low to overcome the poor transmission of microscope objectives at infrared wavelengths (Keatings *et al.,* 2008). Increasing the output power of an OPO, even with careful thermal management, is not straightforward. Nonlinear crystals typically have much lower damage thresholds than laser gain material (Boyd, 2008) and this limits the peak intensity that can be used to pump a femtosecond-pulsed OPO. Therefore, in spite of the very high peak intensities available from pump lasers, only a fraction may be applied in single-pass pumped OPO systems.

We report the development of a novel bi-directional synchronously pumped femtosecond-pulsed OPO system for 3PLSM. This system provides a much higher peak output power than the conventional single-pass pumped OPO geometry, and hence is ideally suited to compensate for the high optical power loss of laser scanning microscopes at long wavelengths. Furthermore, by using an inexpensive fibre laser as the pump source, the total cost of our system, including laser, OPO and microscope, is less than that for a femtosecond-pulsed Ti:Sapphire laser alone. We have applied the system to 3PLSM imaging of auto-fluorescence in live plant cells, specifically those of the alga *Spirogyra*, and compare performance with 2PLSM imaging using a standard, commercially available femtosecond-pulsed Ti:Sapphire laser.

## Experiment

### The laser system

**Fig. 1:**
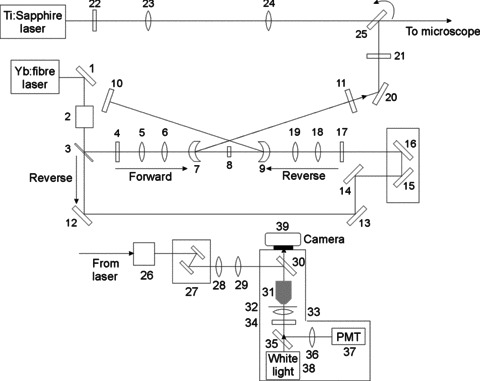
A schematic diagram of the OPO system and Ti:Sapphire beam paths, and the microscopy platform. Elements 1 and 12–16 are highly reflecting plane mirrors at the pump wavelength of λ= 1064 nm. 2 is a Faraday isolator. 3 is a 50/50 thin-plate beamsplitter at λ= 1064 nm. 4 and 7 are half-wave plates at λ= 1064 nm. 5, 6, 18 and 19 are mode-matching lenses. 7 and 9 are zero lens ROC = 100 mm mirrors (zero lens mirrors have the same radius of curvature on both surfaces, so a beam propagating through the element is not focused or defocused, making it easier to design the optical system and control the beam), highly transmitting at λ= 1064 nm and highly reflecting at λ= 1500 nm. 8 is a 3 mm long PPLN crystal. 10 and 20 are high reflectivity plane mirrors at λ= 1500 nm, and 11 is a 50% reflectivity mirror at λ= 1500 nm, which serves as the OPO output coupler. 21 is a λ= 1400 nm long pass filter. 22 is an attenuator for use at λ= 780 nm, and 23 and 24 are lenses to shape the output of the Ti:Sapphire laser to match the profile of the OPO beam. 25 is a high-reflectivity plane mirror at λ= 1500 nm, placed in a flip mount to enable easy switching between the λ= 780 nm and λ= 1500 nm beams. Item 26 is a periscope comprising two broadband infrared reflecting mirrors, and 27 is the scanning system, comprising two galvo mirrors. Item 28 is the *f*= 80 mm scan lens and item 29 is the *f*= 160mm tube lens. 30 is a beam-steering mirror and 31 is the 40x/1.3 N.A. objective lens. 32 is the specimen and/or specimen plane. 33 is the condenser lens and 34 is a filter holder containing the λ= 700 nm short-pass and λ= 629 ± 26.5 nm bandpass filter. 35 is a switch-in reflector, used to send the transmitted fluorescence signal to a collecting lens (36) and then the photomultiplier tube, element 37. 38 is a white-light source, used to illuminate the specimen for bright-field images, captured by 39, a 5 megapixel CMOS camera.

A schematic diagram of the OPO system and microscopy platform is shown in Figure 1.

Full details on the theory and operation of the bi-directional pumped OPO system are available (Norris & McConnell, 2010), and hence we present only a brief overview of the experimental geometry here.

We used a continuous wave mode-locked Yb-doped fibre laser, with an average power of 2W and repetition rate of 80 MHz, pulses of 260 fs duration at a wavelength of λ= 1064 nm and with a spectral width of 12 nm (FWHM) (Femtopower 1060–2-s, Fianium, Southampton, United Kingdom). By blocking the pump beam between elements 3 and 12, we refer to the direction of pumping as the ‘forward’ direction, or by blocking the pump between elements 3 and 4, it is referred to as the ‘reverse’ direction. This is as per a traditional single-pass pumping scheme. With all beam paths open and unblocked, bi-directional pumping, i.e. using both the forward and reverse pass pump beams, was possible.

In order to keep the peak intensity of the pump source below the damage threshold intensity of the nonlinear crystal, only 50% of the average power from the pump was used in a single-pass in both the forward and reverse directions. By using a 50/50 thin-plate beam-splitter (element 3), 1 W of average power was therefore provided in both the forward and reverse pumping directions.

The polarizations of both pump beams were independently controlled by half-wave plates (elements 4, 17) designed for use at the pump wavelength of λ= 1064 nm. Both pump beams were then mode-matched to the linear OPO cavity using two sets of anti-reflection coated spherical lenses (elements 5, 6, 18, 19). The lenses were used to provide a pump beam radius of 20 um in both the forward and reverse direction, which was determined by consideration of the Boyd-Kleinman focusing parameter (Boyd, 2008).

Elements 15 and 16 were highly reflective dielectric mirrors at the pump wavelength, which were mounted on a single translation stage. This created a time delay, permitting temporal displacement of the pump pulse in the reverse direction relative to the circulating signal wavelength pulse. This was used to ensure synchronicity of the system in both forward and reverse directions. Additionally, mirror 10 was placed on a translation stage to control synchronization of resonating signal wavelength pulses.

A 3 mm long periodically poled lithium niobate (PPLN) crystal (element 8) was designed to provide maximum output power at an OPO signal wavelength of λ= 1500 nm. Wavelength tuning of the OPO was possible from λ= 1400 nm to λ= 1650 nm by using the range of periods available in the PPLN crystal and also by adjusting the temperature of the PPLN, but a fixed wavelength of λ= 1500 nm was used for our experiments as this gave the highest intensity fluorescence signal from the *Spirogyra* specimen. All of the mirrors in the OPO resonator were designed to be highly reflective at the signal wavelength and highly transmitting at pump and idler wavelengths, with the exception of the output coupler (element 11), which had a reflectivity of 50% at the signal wavelength. Elements 7 and 9 were zero lens mirrors with a radius of curvature of 100 mm, with the cavity designed to provide a spot size of 19 um inside the PPLN crystal. Zero lens mirrors have the same radius of curvature on both surfaces, so a beam propagating through the element is not focused or defocused, making it easier to design the optical system and control the beam.

The PPLN damage threshold intensity was experimentally demonstrated to be 4 GW/cm^2^, which, when considering the pump source described previously, corresponded to a maximum single-pass pump average power of 1.1 W and a peak power of 49 kW. The PPLN crystal was anti-reflection coated at the pump wavelength on one surface only, namely the surface closest to element 9, due to the crystal being a section cut from a longer PPLN crystal for this study. Neither crystal surface was anti-reflection coated at the signal or idler wavelengths. This resulted in a substantial optical loss and which was therefore detrimental to the overall conversion efficiency. The crystal was kept in a home-made oven at a temperature of 140°C to ensure fixed signal wavelength operation.

The key output properties of the OPO for forward-pass, reverse-pass and bi-directional pumping are shown in Table 1. An average output power of 390 mW was obtained using the bi-directional pump geometry, compared to a maximum of 200 mW when using the single-pass geometry. This increase arises from the linear summation (120 mW + 200 mW) of the independent pump sources and the nonlinear increase of 22% (70 mW) in average power when two counter-propagating independent pump beams of 1 W average power were synchronized with the circulating signal wavelength pulse. The pulse duration did not vary significantly in changing from single-pass to bi-directional pumping of the OPO, and the repetition rate and *M*^2^ value did not change, remaining at 80 MHz and *M*^2^ ∼1.1, respectively.

**Table 1:** Key output properties with the different OPO pump geometries

	Average power (mW)	Pulse duration (fs)	Peak power (kW)
Forward-pass pumping	200	280	7.86 kW
Reverse-pass pumping	120	280	4.71 kW
Bi-directional pumping	390	260	16.54 kW

The output of the OPO (either single-pass or bi-directional) was then coupled into a home-built laser scanning microscope platform. This is shown in the lower half of Figure 1. Element 21 was a band-pass filter (FB1500–12, Thorlabs, Ely, United Kingdom), used to ensure that residual pump wavelength radiation at λ= 1064 nm was blocked and only the signal wavelength output at λ= 1500 nm served as the excitation source for 3PLSM.

Two mirrors and a periscope system were used to steer the signal wavelength radiation towards the scanning system. The scan head module was mounted on an elevated breadboard at the back of the microscope.

The scan head comprised of two galvanometer-driven mirrors mounted at right angles to one another. The 5-mm XY mirror set, XY mount bracket, galvanometer scanners, MicroMax 671XX series driver circuit boards to control the galvanometers and interconnect cables were obtained from Cambridge Technology, Lexington, Massachusetts, U.S.A. This system was capable of speeds of up to 1 kHz and scan angles of up to ± 20°. The mirrors used a protected silver coating on a fused silica substrate with a flatness of λ/4. The XY mount bracket was placed on an XY translation stage for optimum positioning of the mirrors relative to the excitation beam. The power supply units for the driver circuit boards were obtained from XP Power GmbH, Bremen, Germany.

The output beam from the scanning was focused through an achromatic doublet with a focal length of *f*=+80 mm (80 DQ 25, Comar, Cambridge, United Kingdom) serving as the scan lens. The scan lens was positioned 80 mm from the midway point between the scanning mirrors on one side and 80 mm from the intermediate image plane of the tube lens on the other side. The tube lens was an achromatic doublet with a focal length of *f*=+160 mm (160 DQ 25, Comar, Cambridge, United Kingdom). This element was placed 160 mm from the intermediate image plane and therefore 240 mm from the scan lens on one side and 160 mm from the back aperture of the objective lens. The scan lens and tube lens combination doubled the beam width so that the back aperture of the objective lens was consistently overfilled. We used a 40x/1.3 NA objective lens (S Fluor, Nikon, United Kingdom) in the excitation path, and a NA = 0.9 condenser lens in the transmission path. In these experiments, we used a single λ= 700 nm short-pass filter (et700sp-2p8, Bellows Falls, Vermont, USA) and λ= 629 ± 26.5 nm bandpass filter (FF01–629/53–25, Laser 2000, Ringstead, United Kingdom) in the transmission path to block the excitation wavelength and any harmonic generation, such that only auto-fluorescence from the specimens was transmitted. A photomultiplier tube (RFI-QL-30F, Thorn EMI, London, United Kingdom) was employed to collect the fluorescence signal. To control the scanning galvo mirrors and for image capture, we used the freely available MPScope software developed by (Nguyen *et al.,* 2006). For all experiments, the image size was 512 × 512 pixels taken at 2.18 frames per second, with a pixel dwell time of 1.4 μs.

### Live cell specimens

To test our system with a living cellular specimen, we imaged preparations of *Spirogyra*, a eukaryotic green alga with cylindrical cells joined end-to-end to form a filament. The advantage of this material is that every cell and even individual cytoplasmic organelles within it can be imaged clearly. The cylindrical cell shape is maintained by a cell wall containing pectin and cellulose. In *Spirogyra*, the cytoplasm exists as a thin layer between the cell wall and a central vacuole, and ribbon-like chloroplasts are present within the cytoplasm and arranged in a characteristic helical form. The single-photon excitation and emission auto-fluorescence properties of chlorophyll are well-known and understood, with an excitation maximum of around λ= 450 nm and peak emission wavelength of λ= 680 nm, depending on whether the chlorophyll is type a or b (Thorne *et al.,* 1977; Krause & Weis, 1991). *Spirogyra* filaments were mounted in water and placed between a slide and a coverslip for imaging, using silicone grease as a gas-permeable sealant to retard evaporation and fix the coverslip to the slide. With this specimen, we performed 3PLSM using both the single-pass and bi-directional pumped OPO geometries to excite auto-fluorescence, and bright-field transmission imaging to monitor cellular viability.

As a measure of cell viability, we recorded active movement of organelles via cytoplasmic streaming (Allen & Allen, 1978; Shimmen & Yokota, 2004) at 5 min intervals using conventional brightfield imaging and a CMOS camera (GXCam-5, GX Optical, Haverhill, United Kingdom), and recorded any structural changes to the cell following irradiation for 2PLSM and 3PLSM. The 5 min duration was chosen only to represent the duration of a routine imaging experiment. Cytoplasmic streaming is unique to plant cells while mammalian cells use microtubules to transport vesicles. In living plant cells, cytoplasmic organelles are visible in active movement, easily distinguished from Brownian motion by its directional nature, with groups of organelles frequently visible progressing in tandem along defined tracks. The organelles appeared spherical or capsule-shaped and probably included proplastids and mitochondria, which could be seen in clearer contrast if the condenser aperture of the microscope was closed down substantially. It is known that in such cells, streaming occurs at the expense of endogenous cellular energy reserves, and is a good measure of cell health (Allen & Allen, 1978).

### Comparison between the OPO and a Ti:Sapphire laser source

Using the *Spirogyra* specimen, we also compared the performance of the bi-directional OPO for 3PLSM with a femtosecond-pulsed Ti:Sapphire laser (Mira 900-F, Coherent, Santa Clara, California, U.S.A.) of the kind most frequently used in 2PLSM. This experimental geometry is included in the upper half of Figure 1, with the same microscope used as shown in the lower half of Figure 1, with no modification to the microscope. All optical properties of the lasers were identical where possible, including, importantly, average and peak power at the specimen plane (element 8). The Ti:Sapphire was operated at a wavelength of λ= 780 nm, and had a repetition rate of 76 MHz and was arranged to provide a similar beam diameter to the TEM00 OPO to fill the back aperture of the objective lens. The λ= 780 nm wavelength was chosen to avoid light-induced streaming that is reported to occur at wavelengths λ < 780 nm (Plieth & Hansen, 1992). Light is known to induce streaming and produce chloroplast movements in many green plants. It has been suggested that light-induced cytoplasmic streaming occurs because of a change in the availability of ATP (adenosine triphosphate) which is supplied by photosynthesis (Tagaki *et al*., 1985).

Before our imaging experiments, we measured the transmission of the laser scanning microscope (after element 24 to the specimen plane) for both the 3PLSM and 2PLSM geometries. In the case of the 3PLSM system using the OPO at λ= 1500 nm, the transmission was 10% and for the 2PLSM system using the Ti:Sapphire at λ= 780 nm, the transmission was 11%. The average and peak powers shown in Table 1 were therefore scaled to ensure the same average and peak power at the sample when using both the OPO and the Ti:Sapphire laser. Bearing in mind the long (>200 fs) durations of the pulse durations used we have assumed that the higher-order dispersion effects in the microscope system are negligible and equivalent for the two wavelengths used.

## Results

The maximum obtainable average powers for the forward-pass, reverse-pass and bi-directional pumped OPO geometries at the signal wavelength of λ= 1500 nm were measured to be 20 mW, 12 mW and 39 mW at the sample plane, respectively.

Using 200 nm diameter FITC beads, we measured at 780 nm a lateral resolution of 420 nm and an axial resolution of 1400 nm, and at 1500 nm a lateral resolution of 463 nm and an axial resolution of 1530 nm. This fits with the theory of Gu (Gu, 1996): in our case, the longer wavelength balances the resolution improvement in moving from two-photon excitation to three-photon excitation.

**Fig. 2:**
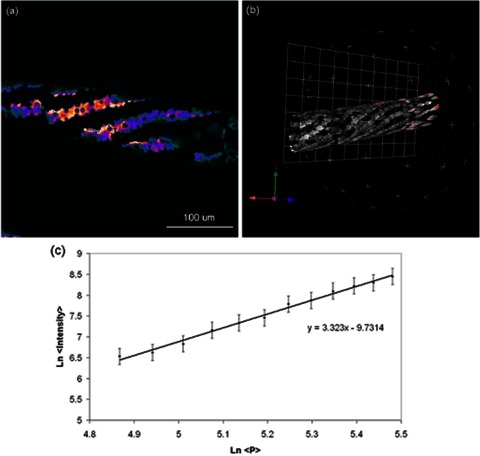
(a) A pseudo-colour cross-sectional auto-fluorescence image of *Spirogyra* taken using the bi-directional pumped OPO at λ= 1500 nm, and with an average power of 31mW at the specimen. The bright bands with serrated edges are optical cross sections of the chloroplasts. The dark circular spots visible at intervals along the chloroplasts are pyrenoids (carbohydrate reserve granules characteristic of this genus of alga). (b) Three-dimensional auto-fluorescence reconstruction of *Spirogyra* using a ‘red hot’ look-up table (volocity, improvision). The unit cell of the grid is 28.03 μm. (c) Log-log plot with a gradient of 3.3 confirms the three-photon nature of the auto-fluorescence excitation.

Figure 2(a) shows 3PLSM images of *Spirogyra* using the bi-directional pumped OPO. With the maximum average power from the single-pass geometry, no useful fluorescence signal was detected. However, with a minimum average power of 27 mW from the bi-directional pumped OPO, three-photon excitation of auto-fluorescence was possible. Figure 2(b) shows a three-dimensional auto-fluorescence reconstruction of the *Spirogyra* chloroplast, obtained using an average power of 31 mW at the specimen. The plot in Figure 2(c) confirms the three-photon nature of the auto-fluorescence excitation process, with a gradient of 3.3 measured from *n*= 5 regions at *n*= 12 power levels. Since it is known that plant cells can scatter a third harmonic when irradiated at these wavelengths (Millard *et al*, 1999), a barrier filter at the appropriate lower bandpass (500 ± 25 nm) was tested and it was established that the chloroplast-specific emissions in the red spectral region were auto-fluorescence, not third harmonic. We note that the auto-fluorescence emission intensities for both two-photon and three-photon imaging were comparable for similar input powers used.

**Fig. 3:**
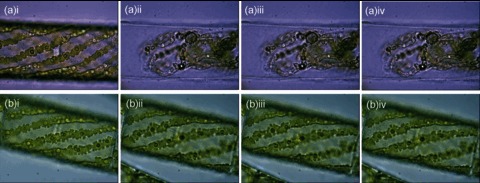
(a) Brightfield images of *Spirogyra* taken after constant irradiation with the femtosecond-pulsed λ= 780 nm Ti:Sapphire laser for time periods of (i) *t*= 0 min, (ii) *t*= 5 min, (iii) *t*= 10 min and (iv) *t*= 15 min. (b) shows brightfield images for the same irradiation times using the femtosecond-pulsed bi-directionally pumped OPO at λ= 1500 nm. The difference in colour is merely due to a different white balance setting of the camera.

Figure 3 presents data from a comparison of the Ti:Sapphire at a wavelength of λ= 780 nm for 2PLSM and the bi-directional pumped OPO at a wavelength of λ= 1500 nm for 3PLSM. The bright-field images are shown from time *t*= 0 min in 5 min increments to 15 min for continuous irradiation with (a) the Ti:Sapphire and (b) the OPO system. At time *t*= 0 min, i.e. prior to irradiation, both specimens appeared healthy and cytoplasmic organelle streaming was evident in brightfield. This was confirmed by capturing movies at 8 frames per second, using the brightfield transmission image. The initial velocity of the organelles at *t*= 0 was measured to be 12.7 μm ± 1.8 μm for the sample subsequently irradiated with the Ti:Sapphire laser, and 13.6 μm ± 2.7 μm for the sample later irradiated with the bi-directional pumped OPO. At time *t*= 5 min of constant irradiation, the sample imaged using the Ti:Sapphire for 2PLSM showed significant structural damage to the chloroplasts within the cytoplasm, and no cytoplasmic organelle streaming was visible. By comparison, even after *t*= 15 min of constant irradiation, the sample imaged using 3PLSM using the OPO at λ= 1500 nm remained structurally unchanged and cytoplasmic organelle streaming continued unabated.

**Fig. 4:**
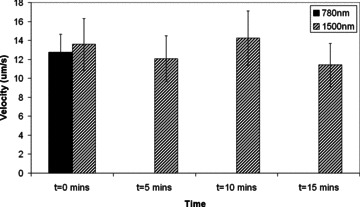
Cytoplasmic organelle streaming velocities in *Spirogyra* from *t*= 0 min to *t*= 15 min were measured from movies taken at 5 min intervals after irradiation with the λ= 780 nm Ti:Sapphire or bi-directionally pumped OPO at λ= 1500 nm (*n*= 5 cells per time interval for each laser).

Using ImageJ for analysis and assuming negligible particle movement in the z-direction, the cytoplasmic organelle streaming velocities from *t*= 0 min to *t*= 15 min were measured. These data are shown in Figure 4 for *n*= 5 organelles. We note that using the λ= 1500 nm OPO, cytoplasmic organelle streaming velocities are near-constant over the *t*= 15 min irradiation period, with a mean velocity over *t*= 15 min of 12.8 μm/s.

## Discussion

Water absorption is often cited as a limiting factor in the use of long wavelength excitation sources for laser scanning microscopy. To evaluate the effect of our system on water absorption, we repeated the method of Schoenle and Hell (Schoenle & Hell, 1998) to calculate the temperature increase at the focal volume of a volume of water. In the case of femtosecond pulses, the irradiation time approaches zero and so a Gaussian model can be used. We assumed an average power at the specimen of *P_av_*=35 mW, a pulse duration in the femtosecond domain with a repetition rate of *f*= 80 MHz and a wavelength of λ= 1500 nm, focused using an objective lens of NA= 1.3, as in our experiment. We assumed an infinite specimen of water, with a uniform extinction coefficient of 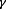
= 10.8 cm^−1^ (Yelin *et al.,* 2002), volume heat capacity *c*_*v*_= 4186 kJ m^−3^ K^−1^, heat conductivity 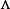
= 0.6 W K^−1^ m^−1^. The effective increase in temperature is given by


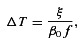
(1)

where, with *k* the wave-vector 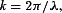



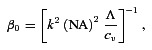
(2)

and


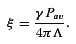
(3)

For our system, *β_0_*= 235.3 ns and 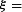
5.013 K. We therefore calculate the effective temperature increase in water at the focal point of our system when using an objective of NA= 1.3 as 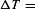
0.266 K. This is around two orders of magnitude higher than for a Ti:Sapphire laser operating at a wavelength of λ= 780 nm but the temperature increase from the bi-directional pumped OPO at a wavelength of λ= 1500 nm is nevertheless unlikely to induce damage by water absorption and heating. Although a temperature increase of two orders of magnitude sounds alarming at first, it is worth remembering that this is an increase in real terms of less than 0.2 K.

As did Schoenle and Hell in the case of the Ti:Sapphire laser, we conclude that the OPO at a wavelength of λ= 1500 nm is unlikely to disturb biological phenomena through the heating of water. However, we must remember that live cells are not entirely composed of water, and we must consider the light-matter interaction with proteins, lipoproteins and endogenous pigments. In our study with *Spirogyra*, it is interesting to note that in Figure 3(a)i–iv, whereas the structural damage to the chloroplasts is evident, there is no change in the cell wall. We therefore conclude that the photo-damage observed after irradiation with the Ti:Sapphire is not a consequence of water absorption, but absorption by other molecules present within the chloroplasts, presumably chlorophyll or carotenoid pigments which have strong absorption bands in the visible range, but very low absorption in the nearinfrared.

The fact that the cytoplasmic streaming is not compromised by radiation offers the possibility for studies in alternative specimens. We note the work of Sheetz *et al*., where cytoplasmic streaming in single cells of *Nitella*, a characean alga, was studied using myosin-coated bead labelling (Sheetz *et al.,* 1984). We were able to excite three-photon fluorescence from FITC-labelled beads at the same intensities used for the imaging of *Spirogyra*. Our laser therefore offers the opportunity of extending the work of Sheetz *et al*., providing improved three-dimensional information and with potentially much better longevity of the motor proteins of the cell. If cytoplasmic streaming is unaffected, it seems likely that this laser could be used also in studies of exo- and endo-cytosis and other forms of motility, with the added value over other methods of the optical sectioning inherent in the multiphoton process. Because cytoplasmic streaming in this form is more obvious in plant cells, alternative means of assessing cell viability (e.g. permeability to propidium iodide or similar dyes) might be preferable for studies of mammalian cells.

## Conclusion

In summary, we have reported the application of a bi-directional pumped optical parametric oscillator with signal wavelength output at λ= 1500 nm for 3PLSM.

Using the higher average and peak powers from this bi-directional OPO, we overcame the >70% optical loss in the infrared spectral region observed in the laser scanning microscope and objective lenses that renders these systems otherwise difficult or impossible to use for long wavelength excitation imaging. Furthermore, by using an inexpensive fibre laser as the pump source rather than a Ti:Sapphire, the total cost of our system, including laser, OPO and microscope, is almost an order of magnitude less than a commercial Ti:Sapphire, OPO and laser scanning microscope package.

We performed 3PLSM of live *Spirogyra* cells using the bi-directional pumped OPO and compared auto-fluorescence imaging capability with 2PLSM using a standard commercial femtosecond pulsed Ti:Sapphire laser at a wavelength of λ= 780 nm, with care taken to match the peak and average powers from both lasers. With the λ= 780 nm Ti:Sapphire laser, gross structural damage was observed after *t*= 5 min of continuous irradiation, while the λ= 1500 nm bi-directional OPO gave a notable improvement in cell viability: it had no effect on the structure of the chloroplasts, and cytoplasmic organelle streaming velocities were preserved over *t*= 15 min of constant irradiation.

The increased power available from the bi-directional pumped OPO described here, coupled with its innocuous effect on living cells, may well prove of great value, not just in the study of plant cells. Photo-damage is the chief limitation of laser scanning microscopy of living cells, and our work suggests a new way of mitigating it.

The lack of damage of the long-wavelength beam under imaging conditions warrants a survey of the imaging properties of this laser with diverse materials, including tissue culture cells, embryos, including those of mammals, which are particularly sensitive to short wavelengths (Squirrel *et al.,* 1999), and with important molecular probes such as photoproteins and well-known fluorochromes. We are already engaged in such a survey.
